# The Story of the Insane from Year to Year

**Published:** 1906-10-13

**Authors:** 


### THE STORY OF THE INSANE FROM YEAR TO YEAR.

Eighteenth Annual Series.

### JOINT COUNTIES' ASYLUM, CARMARTHEN.

At the beginning of the year this asylum contained 674 patients, and at the end of the year only 660 were in residence. There were 92 first admissions, 13 readmissions, 44 recoveries, 6 discharged relieved, 11 discharged not improved, and 57 died. The total number of treatments was 779, and the average daily number resident was 664. The recoveries were 45.8 per cent. of the admissions, and the deaths were 8.56 per cent. of the average number resident. Of the year's deaths 14 were caused by cerebral and spinal diseases, 4 by consumption, and 4 by pneumonia. Of the year's admissions 20 owed their insanity to moral causes, such as domestic trouble and religion, while among the physical causes intemperance in drink accounts for 9, hereditary influence for 23, and previous attacks for 17. When referring to the admissions, Dr. Goodall mentions the case of a woman who has a brother and sister in the asylum and a brother in a neighbouring asylum; of another who has a brother and four cousins in asylums, and he says that should these patients become uncertifiable they must be discharged under the re-certification clause of the Lunacy Act, and it is the same with recurrent cases, who must be discharged, although their readmission is certain; and Dr. Goodall tells us of a female patient who was admitted fourteen times in twenty-one years. The ratepayers have to pay for all these re-certifications and removals, future generations suffer from an increase of insanity, and asylum officers have much useless work to get through, and all because a stupid clause was inserted in the Lunacy Act, a clause which will probably remain, although its drawbacks have been exposed time after time. The Committee place on record "their satisfaction with the way the institution is managed by the Medical Superintendent." The weekly charge per head to the Unions within the counties is 9s., out-county cases pay 14s., and private patients pay from 10s. to 30s. a week.

